# Immunophenotypic Landscape and Prognosis-Related mRNA Signature in Diffuse Large B Cell Lymphoma

**DOI:** 10.3389/fgene.2022.872001

**Published:** 2022-06-08

**Authors:** Yanan Jiang, Huimeng Sun, Hong Xu, Xin Hu, Wenqi Wu, Yangyang Lv, Jinhuan Wang, Su Liu, Yixin Zhai, Linyan Tian, Yafei Wang, Zhigang Zhao

**Affiliations:** ^1^ Key Laboratory of Cancer Prevention and Therapy, Department of Hematology, Tianjin Medical University Cancer Institute and Hospital, National Clinical Research Center for Cancer, Tianjin’s Clinical Research Center for Cancer, Tianjin, China; ^2^ Key Laboratory of Cancer Prevention and Therapy of Tianjin, Key Laboratory of Molecular Cancer Epidemiology, Department of Epidemiology and Biostatistics, Tianjin Medical University Cancer Institute and Hospital, National Clinical Research Center for Cancer, Tianjin’s Clinical Research Center for Cancer, Tianjin, China; ^3^ Department of Oncology, Institute of Urology, Second Hospital of Tianjin Medical University, Tianjin, China

**Keywords:** diffuse large B cell lymphoma, immunotherapy, mRNA expression, tumorinfiltrating lymphocytes, immune risk signature, prognosis

## Abstract

Diffuse large B cell lymphoma (DLBCL) exhibits a tightly complexity immune landscape. In this study, we intended to identify different immune phenotype and to examine the immune related mRNA signature for clinical characteristic, therapeutic responsiveness as well as risk stratification and survival prediction in DLBCL. We identified two immune infiltration subtypes of DLBCL patients based on 28 immune cell types. GSEA analysis uncovered the concordant classification of two robust significant subtypes of DLBCL. Considering the convenient application of the immune infiltration subtypes for prognostic prediction, we developed a risk score based on the differentially expressed genes between the Immunity-H and Immunity-L groups. By a least absolute shrinkage and selection operator (LASSO)-Cox regression model, a sixteen-gene risk signature, comprising ANTXR1, CD3D, TIMP1, FPR3, NID2, CTLA4, LPAR6, GPR183, LYZ, PTGDS, ITK, FBN1, FRMD6, PLAU, MICAL2, C1S, was established. The comprehensive results showed that the high-risk group was correlated with lower immune infiltration, more aggressive phenotypes, lower overall survival and more sensitive to lenalidomide. In contrast, a low-risk group score was associated with higher immune infiltration, less aggressive phenotypes, better overall survival and more likely to benefit from PD-1/PD-L1 inhibitors. Finally, a nomogram comprised of the risk score and IPI score was verified to more accurately predict the overall survival of DLBCL than traditional clinical prediction models. Altogether, our data demonstrate the heterogeneity of immune patterns within DLBCL and deepen our molecular understanding of this tumor entity.

## Introduction

Diffuse large B cell lymphoma (DLBCL) is the most common subtype of lymphoma in adults, accounting for approximately 30% of newly diagnosed non-Hodgkin’s lymphomas (NHLs) annually (Bartlett et al., 2019). Two distinct molecular subtypes, germinal center B cells (GCBs) and activated B cells (ABCs), were identified based on cell of origin (COO) (Alizadeh et al., 2000). The standard frontline therapy for DLBCL patients is cyclophosphamide in combination with doxorubicin, vincristine and prednisone (CHOP) with or without rituximab (R-CHOP), regardless of the subtype. However, one-third of patients will eventually fail R-CHOP treatment (Pfreundschuh et al., 2011; Oki et al., 2014). The International Prognostic Index (IPI) and Revised International Prognostic Index (R-IPI), which are based on five clinical characteristics [stage, age, lactic dehydrogenase (LDH) level, performance status, and extranodal sites involved], are common prognostic and predictive tools for DLBCL (Ziepert et al., 2010). The limitations of IPI and R-IPI are their inability to predict individualized therapy for DLBCL patients. Therefore, great challenges exist regarding how to accurately predict survival and provide individualized treatment recommendations.

More recently, the critical role of the tumor microenvironment (TME) has been widely recognized in tumor initiation, proliferation and subsequent drug resistance, including in lymphoma (Khurana and Ansell, 2020; Merryman et al., 2020). Immune landscapes in lymphoma appear to be heterogeneous and can be categorized as “inflamed” and “noninflamed” or “immune excluded” lymphoma (Ciavarella et al., 2018; Li et al., 2019; El Hussein et al., 2020). In recent years, the landscape for DLBCL immunotherapy strategies has become increasingly.

Here, we performed mocrowded. Novel therapies such as CAR-based cell therapies exhibit the most promising results, particularly for patients who have demonstrated resistance to chemotherapy (Neelapu et al., 2017; Chavez and Locke, 2018; Locke et al., 2019). Similarly, immunomodulatory drugs such as lenalidomide also have a variety of effects on the immune system (Hernandez-Ilizaliturri et al., 2011; Feldman et al., 2014; Garciaz et al., 2016). However, PD1/PD-L1 blockade seems to have unimpressive results (Sheikh and Kuruvilla, 2019; Tomassetti et al., 2019; Frigault et al., 2020). The immune response to cancer is tightly correlated with the tumor microenvironment. A greater understanding of the types and roles of immune cells in the TME will enable us to identify candidate patients who will benefit from targeted immunotherapy and to discover biomarkers with important prognostic significance in DLBCL.lecular subtyping of 738 DLBCL patients from the Gene Expression Omnibus (GEO) and The Cancer Genome Atlas (TCGA) databases. We revealed that DLBCL patients could be classified into the high- and low-immune cell infiltration groups based on 28 immune cell types. Meanwhile, we determine this grouping strategy reflect the distinct immune features of DLBCL itself. Considering the convenient application of the immune infiltration subtypes for prognostic prediction, we developed a risk score based on the differentially expressed genes between these two immune subtypes. Finally, our findings suggest that the risk score is a valuable biomarker for predicting the benefit of immunotherapy. In addition, it was an independent predictor of survival and can improve the prognostic value when combined with IPI.

## Materials and Methods

### Expression and Clinical Datasets

This analysis included 1130 DLBCL patients with available survival data in four cohorts. Three microarray datasets, GSE87371, GSE31312, GSE10846, were derived from GEO database. TCGA-DLBC was extracted from the TCGA program. The “sva” package was used to remove batch effects. The R package “maftools” was used to identify the mutation status of TCGA-DLBC.738 DLBCL patients including TGCA database (*n* = 47)、GSE87371 (*n* = 221) and GSE31312 (*n* = 470) was regarded as the training cohort. GSE10846 (*n* = 392) was regarded as the validation cohort. Characteristics of the study patients for the DLBCL training cohorts and validation cohorts are given in Supplementary Table S1.

### Identification of Gene Expression-Based Subtypes

Subtype classification of DLBCL training cohort was based on Immune infiltration cell-Related Genes using the R package “ConsensusClusterPlus”. 80% item resampling, 100 resamplings, and a maximum evaluated K of six were selected for clustering. The cumulative distribution function (CDF) and consensus heatmap were used to assess the best K. Using the limma R package, we calculate differential gene expression based on RNA-seq counts data of two immune subgroups.

### The Tumor Microenvironment Score of Immune Subgroups

Gene expression levels of various genes including members of Immune complex of the major histocompatibility complex (MHC), immune costimulator checkpoint (ICP), and immune co-inhibitor checkpoint (IAP) was used to assess the differences between the two subtypes. Stromal Score, Immune Score, ESTIMATE Score were calculated using the ESTIMATE R package.

### Gene Sets Enrichment Analysis

We used the R software package “clusterProfiler” for gene set enrichment analysis (GSEA) to study the biological process differences between immune infiltration subtypes and two risk groups. GSEA was conduced based on the expression of all the genes in the two subtypes or risk groups. The results of Kyoto Encyclopedia of genes and genomes (KEGG) and Gene Ontology (GO) are displayed through GSEA plot.

### Construction of Risk Assessment Model

We first get genes that were tightly correlated with prognosis by univariate Cox regression analysis (*p* < 0.001). Then the least absolute shrinkage and selection operator (LASSO) Cox regression algorithm was used to select prognostic immune related signature and calculate variable coefficients with the “glmnet” package. Then, we calculated the risk score of each sample according to the following equation:

Risk score=Exp1∗Coe1+Exp2∗Coe2+Exp3∗Coe3+…Expi∗Coei

where coei is equal to the gene coefficient, and Expi represents the gene expression level. ALL DLBCL patients were divided into high- and low-risk group based on the cut-off value.

### Predict Chemotherapeutic Response

The R package “pRRophetic” (Geeleher et al., 2014a) was employed to predict chemotherapeutic response in DLBCL patients, of which the predicted drug sensitivity of the samples was demonstrated using ridge regression, and the prediction accuracy was assessed using 10-fold cross-validation based on the GDSC training set (Geeleher et al., 2014b).

### Prognostic Value of the Prognostic Signature

Kaplan-Meier survival curves was used to compare the overall survival (OS) of DLBCL patients in the high- and low-risk groups. Receiver operating characteristic (ROC) analysis was used to compare the overall survival (OS) of DLBCL patients in the high- and low-risk groups. Receiver operating characteristic (ROC) curve analysis. was used to assessment the sensitivity and specificity of the Prognostic Signature in predicting OS. Univariate and Multivariate Analysis was used to assess whether clinical features and risk scores are prognostic risk factors. The R package “rms” was used to plot nomogram predict the of 3-, and 5-years OS DLBCL patients.

### Statistical Analysis

Kaplan-Meier analysis with log-rank tests was performed to compare differences in prognosis. *p* < 0.05 was considered statistically significant. Categorical variables were described as percentages. A chi-square test was performed to determine the difference of clinical and molecular parameters between two groups. Univariate and multivariate Cox regression analyses were conducted to determine factors with independent prognostic value. Pearson’s correlation analysis was carried out to compare correlations between two groups. The Wilcoxon test was used to compare differences between two groups of non-normally distributed data. R software (version 4.0.3), SPSS 22.0, and Prism eight were used for statistical analysis and graphing.

## Results

### Molecular Subtypes Related to Immune Infiltration in Diffuse Large B Cell Lymphoma

**FIGURE 1:**
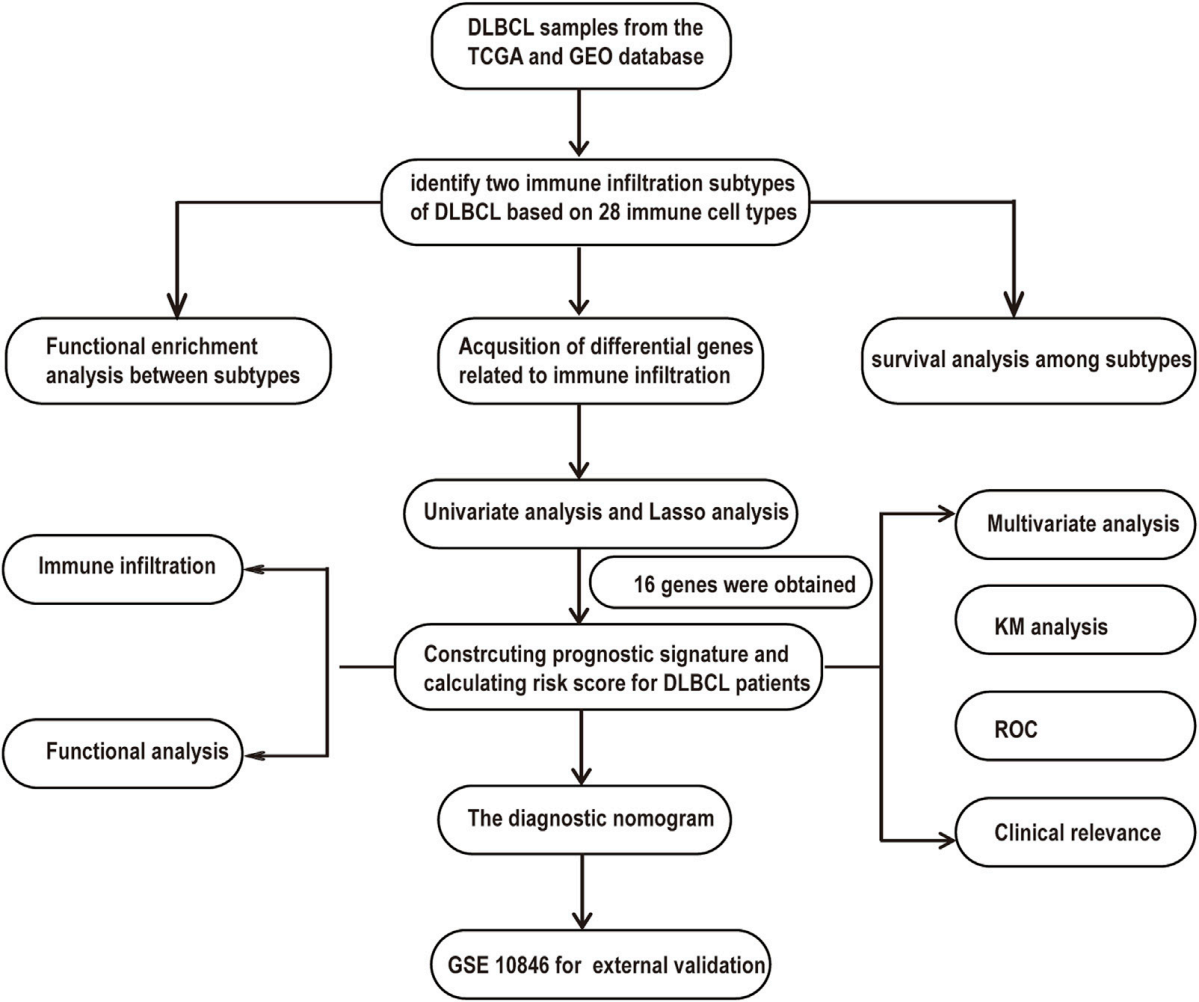
A flowchart for the process of the study.

**FIGURE 2:**
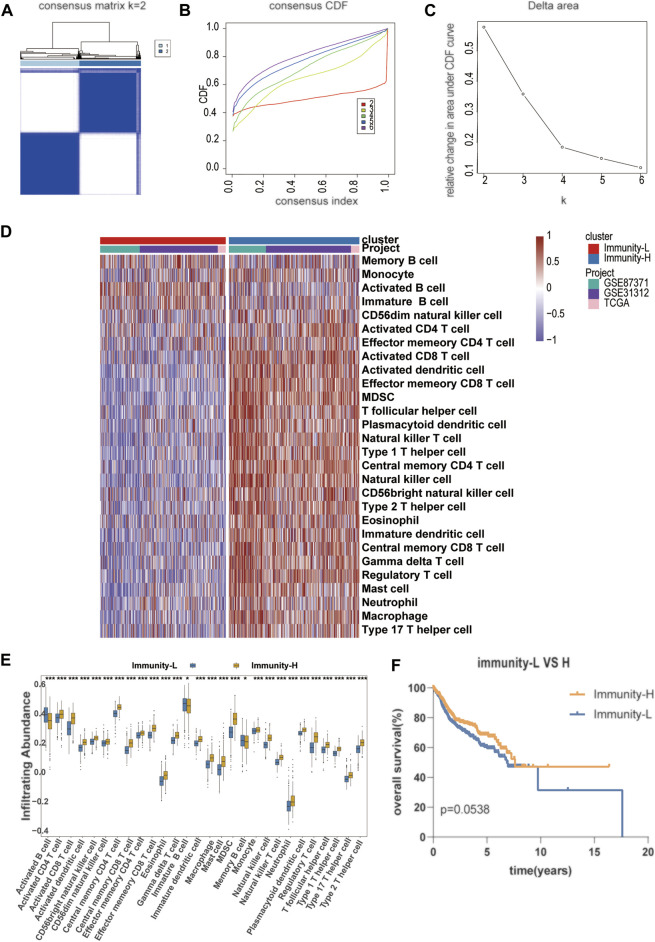
Establishment of a novel molecular immune subgroup **(A–C)** The optimal number of clusters (K = 2) was determined from cumulative distribution function (CDF) curves, and the classification effect is the best **(D)** Immune cell infiltration subtype is shown in the form of a heat map, with the 738 patient samples shown as columns **(E)** The infiltration abundance of different immune cells in Immunity-L and Immunity-H group **(F)** The Kaplan–Meier survival curves with log-rank test between Immunity-H and Immunity-L group.

A flowchart is shown to demonstrate the process of our study (Figure 1). This analysis included 1130 DLBCL patients with available survival data in four cohorts obtained from the TCGA and GEO databases. A total of 738 DLBCL patients, who were included in the TGCA database (*n* = 47), GSE87371 (*n* = 221) and GSE31312 (*n* = 470), were considered the training cohort. GSE10846 (*n* = 392) was considered the validation cohort. The clinical information of DLBCL patients of training and validation cohorts is summarized in Supplementary Table S1. To further stratify the DLBCL patients into different immune infiltration groups, consensus cluster analysis was conducted. The optimal number of clusters was two, which was defined by CDF curves (Figures 2A–C).

An unsupervised hierarchical cluster analysis of DLBCL patients based on immune infiltration-related genes separated DLBCL into high- and low-immune cell infiltration groups. The high immune cell infiltration group (Immunity-H, *n* = 376) was enriched for almost all selected immune cell subtypes. Cases in the low immune cell infiltration group (Immunity-L, *n* = 362) exhibited lower selected immune cell infiltration, except activated B cells (Figures 2D,E). A comprehensive summary of the patient characteristics of the two groups is shown in Supplementary Table S2. Group membership within the two subtypes was associated with similar clinical characteristics, such as age, sex, stage, and COO. The Immunity-H group, with a longer overall survival, contained more patients with lower IPI scores. The Kaplan–Meier survival curves between the Immunity-H and Immunity-L groups are shown in Figure 2F.

### Immune Infiltration Defines a Biologically Distinct Subgroup Within Diffuse Large B Cell Lymphoma

**FIGURE 3:**
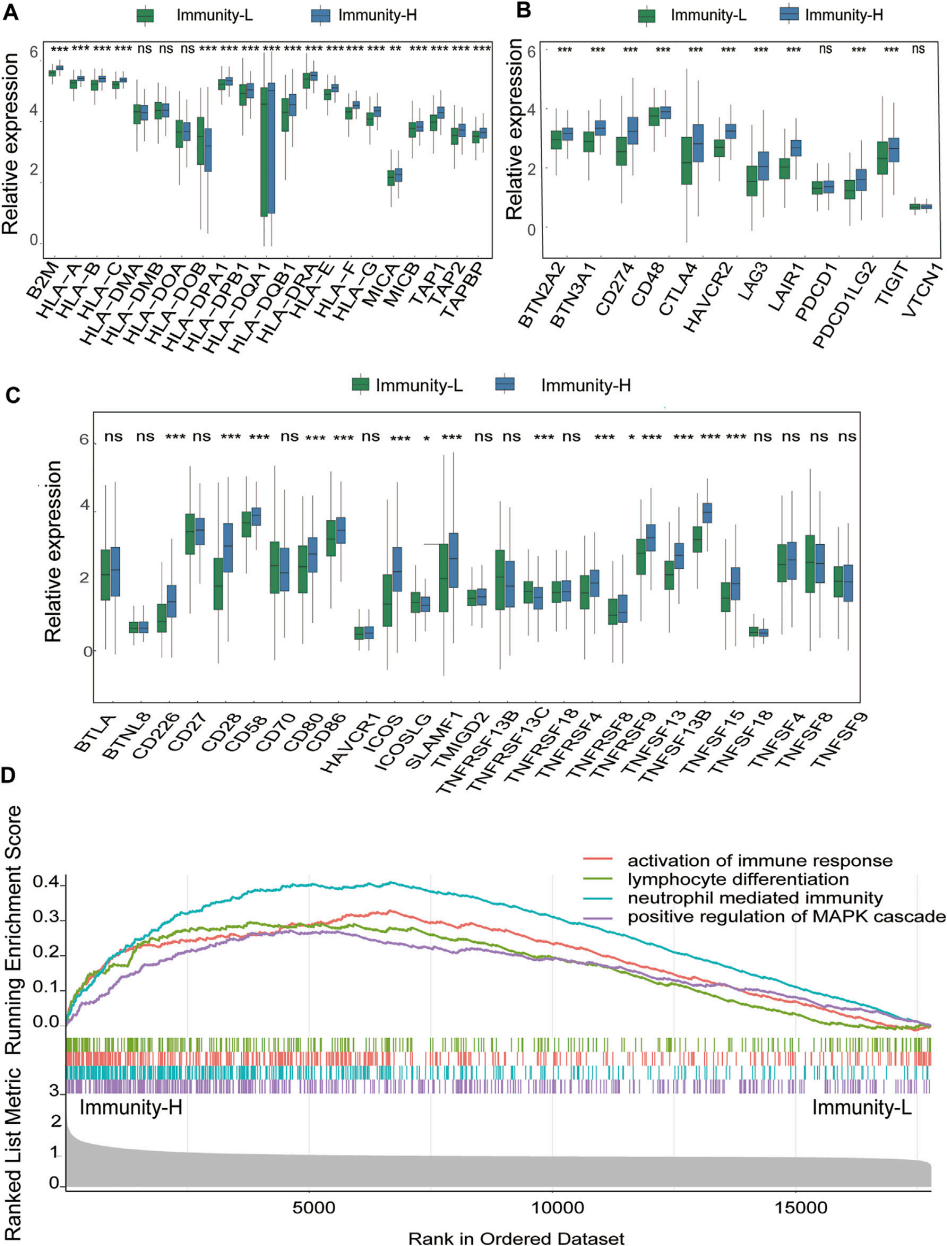
Immune Infiltration Defines a Biologically Distinct Subgroup Within DLBCL **(A–C)** The gene expression level of the gene set, including major histocompatibility complex (MHC), immune co-inhibitor checkpoints (IAP), and immune co-stimulator checkpoints (ICP) were all significantly different in the two immune infiltration subtypes (*p* < 0.05) **(D)** Functional annotation of the two immune infiltration subtypes.

To determine the accuracy of this grouping strategy, we determined the major histocompatibility complex (MHC), immune coinhibitor checkpoint (IAP), and immune costimulator checkpoint (ICP)-related gene expression in the two groups (Figures 3A–C). For most of the related genes, the bar chart analysis showed that there was a significant positive correlation between the Immunity-H group and MHC, IAP, and ICP. These results indicated that impaired immune surveillance of tumor cells is an escape mechanism in the Immunity-L group. Furthermore, we analyzed the functional context of these two subtypes by conducting Gene Ontology (GO). In the GO analysis of the two groups show that Immunity-H group were mainly enriched in immune-related functions, such as activation of immune response, lymphocyte differentiation, neutrophil mediated immunity, positive regulation of MAPK cascade (Figure 3D, false discovery rate <0.05).

### Identification of a Classification-Related Prognostic Signature for Diffuse Large B Cell Lymphoma

**FIGURE 4:**
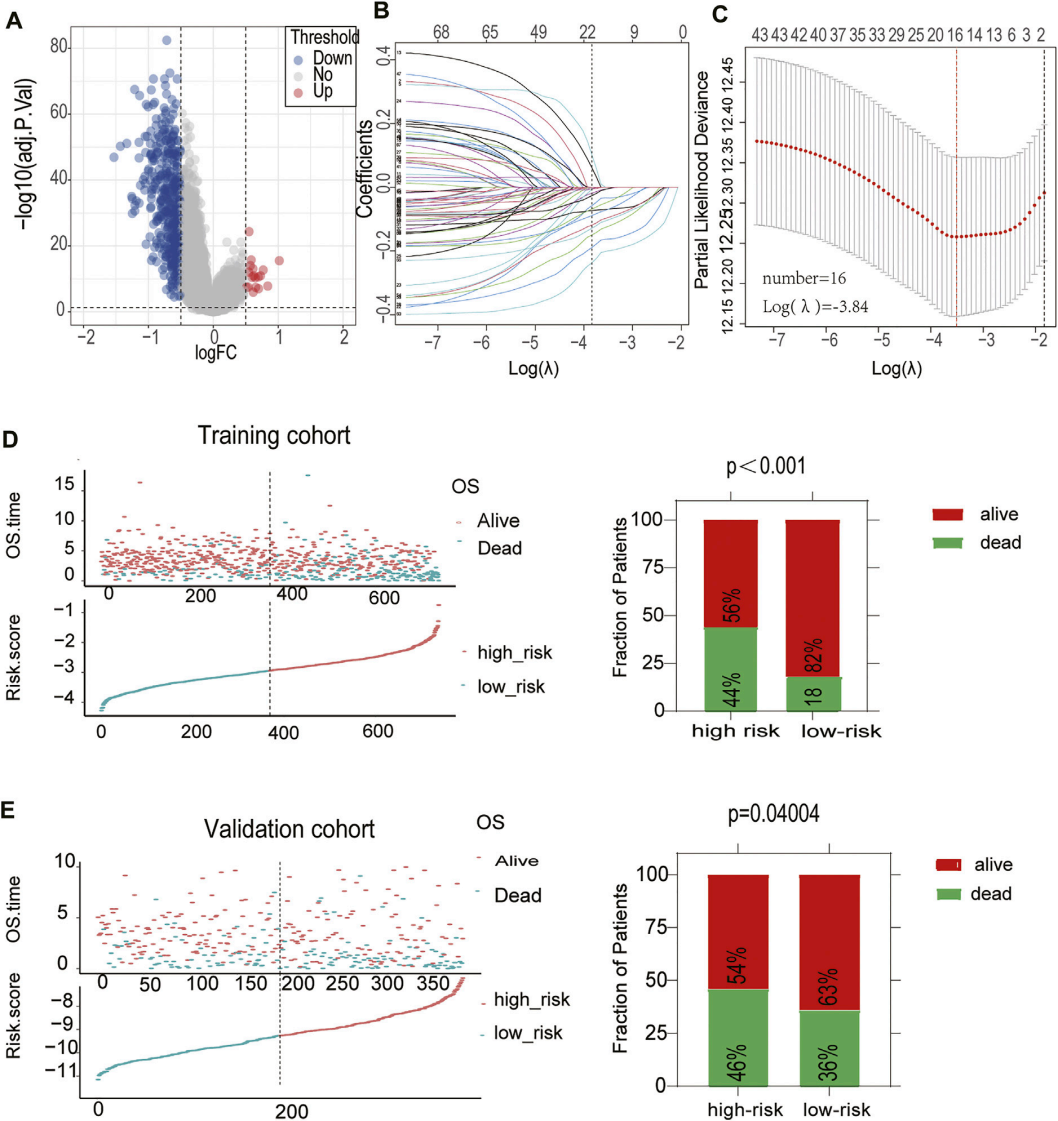
Construction of a prognostic signature for DLBCL **(A)**The volcano plot showed that 18 genes were up-regulated and 471 genes were downregulated between the two immune infiltration subtypes. Each red dot showed an up-regulated gene, and each blue dot shows a down regulated gene (|Log2 Fold Change| >0.5 and FDR <0.05) **(B)** LASSO coefficients of the 69-subgroup related-associated genes. The red vertical line represents the best value based on the minimum criterion, which resulted in 16 nonzero coefficients **(C)** Sixteen genes selected to construct the immune-related genes prognostic signature by LASSO regression analysis. The two dotted vertical lines indicate the minimum and 1-standard error criteria employed to identify the best values. Each curve represents a gene **(D,E)** The distribution of the risk Score and Survival overview of patients in the training and validation cohort.

**FIGURE 5:**
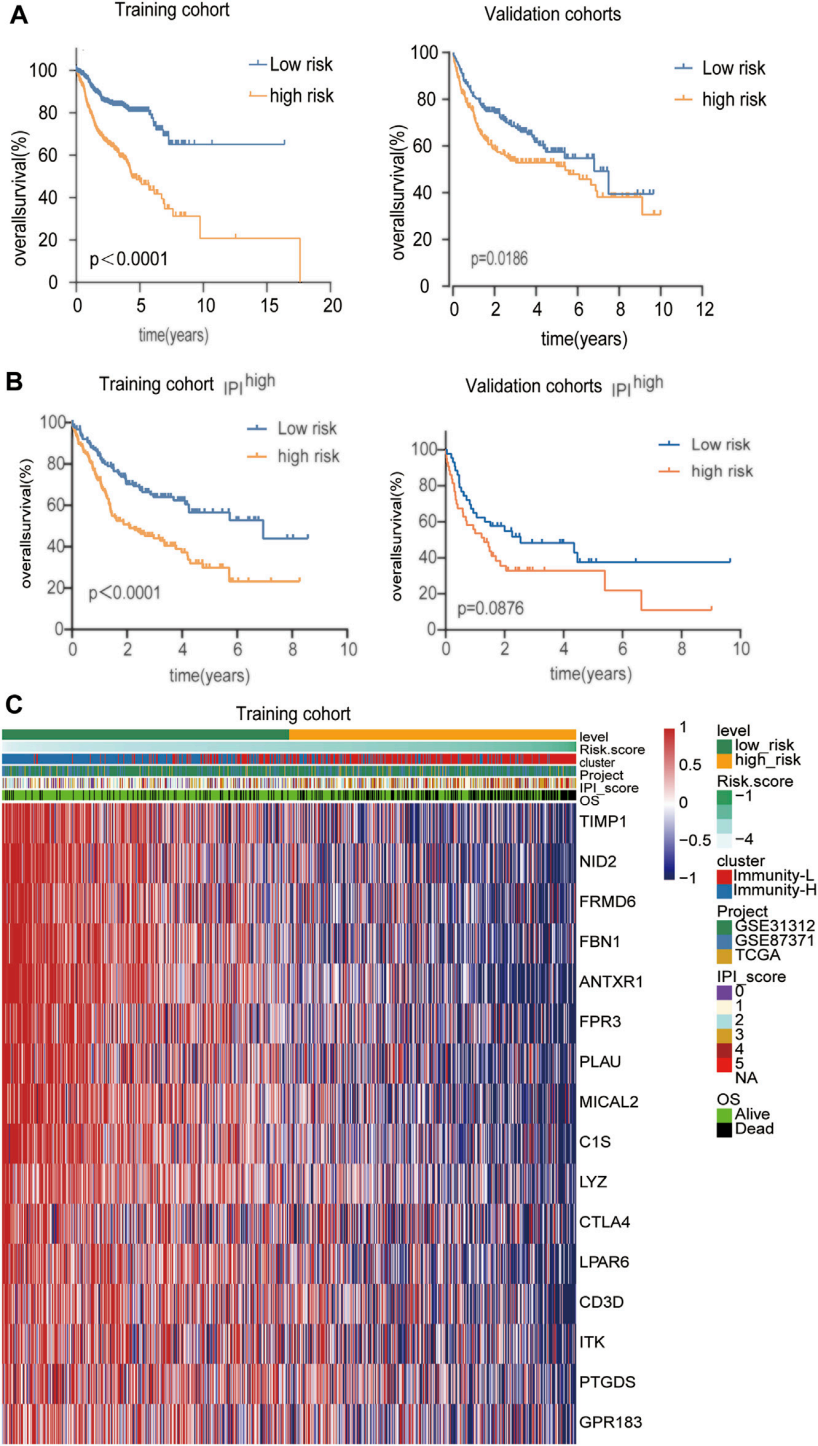
Identification of a classification-related prognostic signature for DLBCL **(A)** Kaplan-Meier analysis with log-rank test of the high versus low risk score for the training and validation cohort **(B)** Kaplan-Meier analysis with log-rank test of the high versus low risk score in the IPI ^high^ group for the training and validation cohort **(C)** The differential expression of the 16 genes in the high- and low-risk groups in the training cohort.

Considering the convenient application of the immune infiltration subtypes for prognostic prediction, we developed an immune risk score based on the differentially expressed genes between the Immunity-H and Immunity-L groups. According to the cutoff thresholds of |Log2 Fold Change|>0.5 and FDR <0.05, a total of 489 mRNAs that were differentially expressed were obtained, of which 18 were upregulated and 471 were downregulated (Figure 4A). We then performed univariate analysis and LASSO analysis to select the gene set with the best prognostic value (Figures 4B,C). A 16-gene signature (ANTXR1, CD3D, TIMP1, FPR3, NID2, CTLA4, LPAR6, GPR183, LYZ, PTGDS, ITK, FBN1, FRMD6, PLAU, MICAL2, C1S) was identified and the risk score of each case was computed with the gene expression level and regression coefficient. The Kaplan-Meier analysis of the 16 immune subtype-related genes is shown in Supplementary Figure S1. Next, patients in training cohorts were assigned to a low-risk or high-risk group based on the median value of risk scores, which was used as the cutoff value. We also calculated the risk scores of patients in the validation cohort with the same coefficients to validate this signature. According to the distribution of the risk score and survival status, we detected the association between this signature and the proportion of deaths (*p* < 0.05). A higher risk score was associated with a higher proportion of deaths (Figures 4D,E) both in the training and validation cohorts. Subsequently, we analyzed the expression of the 16 genes included in the signature in the high- and low-risk groups. To test the value of the risk score for DLBCL, we performed survival analyses. The results showed that the survival time of the high-risk group was significantly shorter than that of the low-risk group in the training and validation cohorts (*p* < 0.05) (Figure 5A). To address the question of whether the risk score based on the newly established molecular immune signature is a unique feature of DLBCL, we tested its role in different IPI scores. We found that the survival time of the high-risk group was also shorter than that of the low-risk group in the IPI ^high^ groups, both in training and validation cohort (Figure 5B). For IPI ^low^ group, the survival time of the high-risk group was significantly shorter than that of the low-risk group, only in training cohort (Supplementary Figures S2A,B). Moreover, Heatmap analysis was used to visualize the expression of the 16 genes in DLBCL patient samples in both the training and validation cohorts (Figure 5C, Supplementary Figure S2C).

### The Prognostic Signature is Related to Immune Infiltration and Clinical Characteristics

**FIGURE 6:**
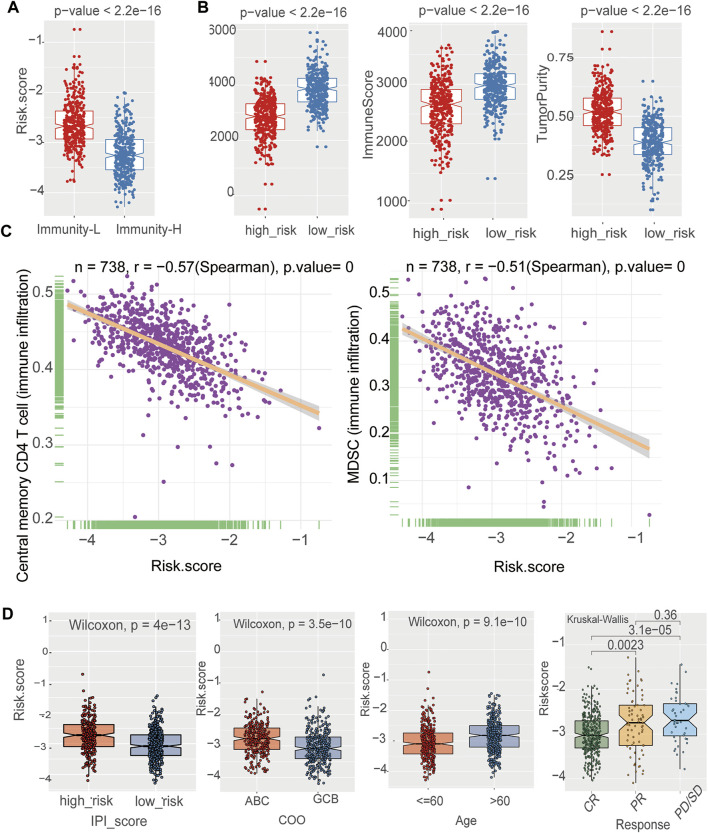
The prognostic signature was related to immune cells and clinical characteristics **(A)** The negative correlation between the risk Score and immune infiltration in the training cohort. Wilcoxon test **(B)** The boxplot showed that there was a statistical difference in Immune Score, ESTIMATE Score, and Tumor Purity between the two immune infiltration subtypes (*p* < 0.05) in the training cohort. Wilcoxon test **(C)** Assessment of immune cell infiltration abundance by the risk Score. There were negative correlation between the risk Score and central memory CD4 T cell 、MDSC in the training cohort **(D)** The box plot shows the risk score for indicated subgroups, with a significantly low risk score for IPI low、age ≤60 years old, GCB, the CR group as compared with other groups in the training cohort.

Notably, the risk score was significantly correlated with immune infiltration. A high-risk score was associated with lower immune infiltration (Figure 6A). To further confirm the immune heterogeneity between these two risk groups, we examined the distribution of stromal and immune content of each group. The risk score was significantly negatively correlated with the immune score and ESTIMATE score (Figure 6B) (*p* < 0.05). Patients in the low-risk group had a lower tumor purity, suggesting that low-risk group patients contained a higher number of immune cells (Figure 6B). Pearson’s analysis was performed to determine the correlations between the risk score and the specific type of each immune cell. Except for activated B cells, a negative correlation was observed between the risk score and immune infiltration cells, such as central memory CD4 T cell and Myeloid-derived suppressor cells (MDSC) (Figure 6C, Supplementary Figure S3). By stratified analysis, we found that the risk score showed excellent prognostic value among subjects with different baseline characteristics. There was a tightly correlation between the risk score and IPI, age (Figure 6D). For high-risk group, there was a higher proportion of patients in the IPI ^high^, age>60 years old groups. Stratified analysis further revealed that the risk score was lower in CR patients than in PR patients, but there were no statistically significant differences between PR and PD/SD patients (Figure 6D). In addition, the risk score was higher in ABC-DLBCL patients than in GCB-DLBCL patients (Figure 6D).

### The Prognostic Signature is Related to the Response to Chemotherapy and Immunotherapy

**FIGURE 7:**
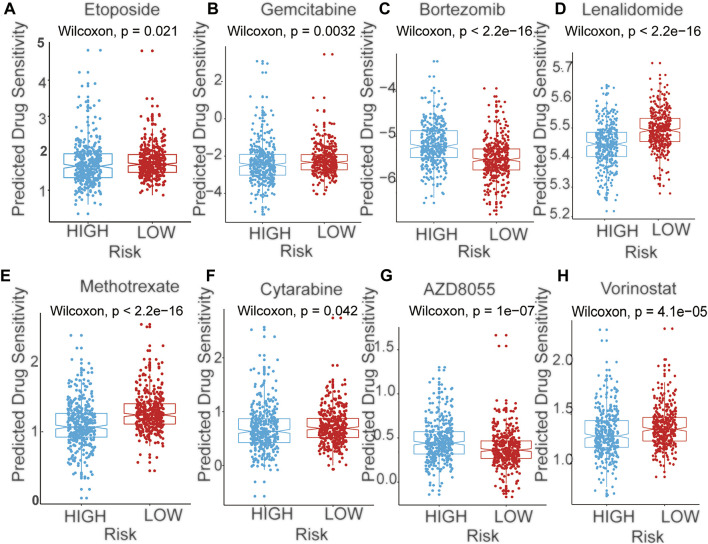
The predicted sensitivity of chemotherapeutic agents with mRNA signature in training cohort **(A)** Etoposide **(B)** Gemcitabine **(C)** Bortezomib **(D)** Lenalidomide **(E)** Methotrexate **(F)** Cytarabine **(G)** AZD8055 **(H)** Vorinostat.

We next performed a prediction analysis of response to therapy in the two-risk group by applying the “pRRophetic” method. There were obvious differences in the risk score and response to different drugs, in both the training and validation cohorts. (Figure 7, Supplementary Figure S4). Patients in the high-risk group were more sensitive than those in the low-risk groups for the following chemotherapy drugs: gemcitabine, lenalidomide, methotrexate, cytarabine, and vorinostat. Conversely, patients in the low-risk group were more sensitive than those in the high-risk groups for the following chemotherapy drugs: bortezomib and AZD8055. (*p* < 0.05).

### The GSEA Enrichment Analysis Related to the Two Risk Group

**FIGURE 8:**
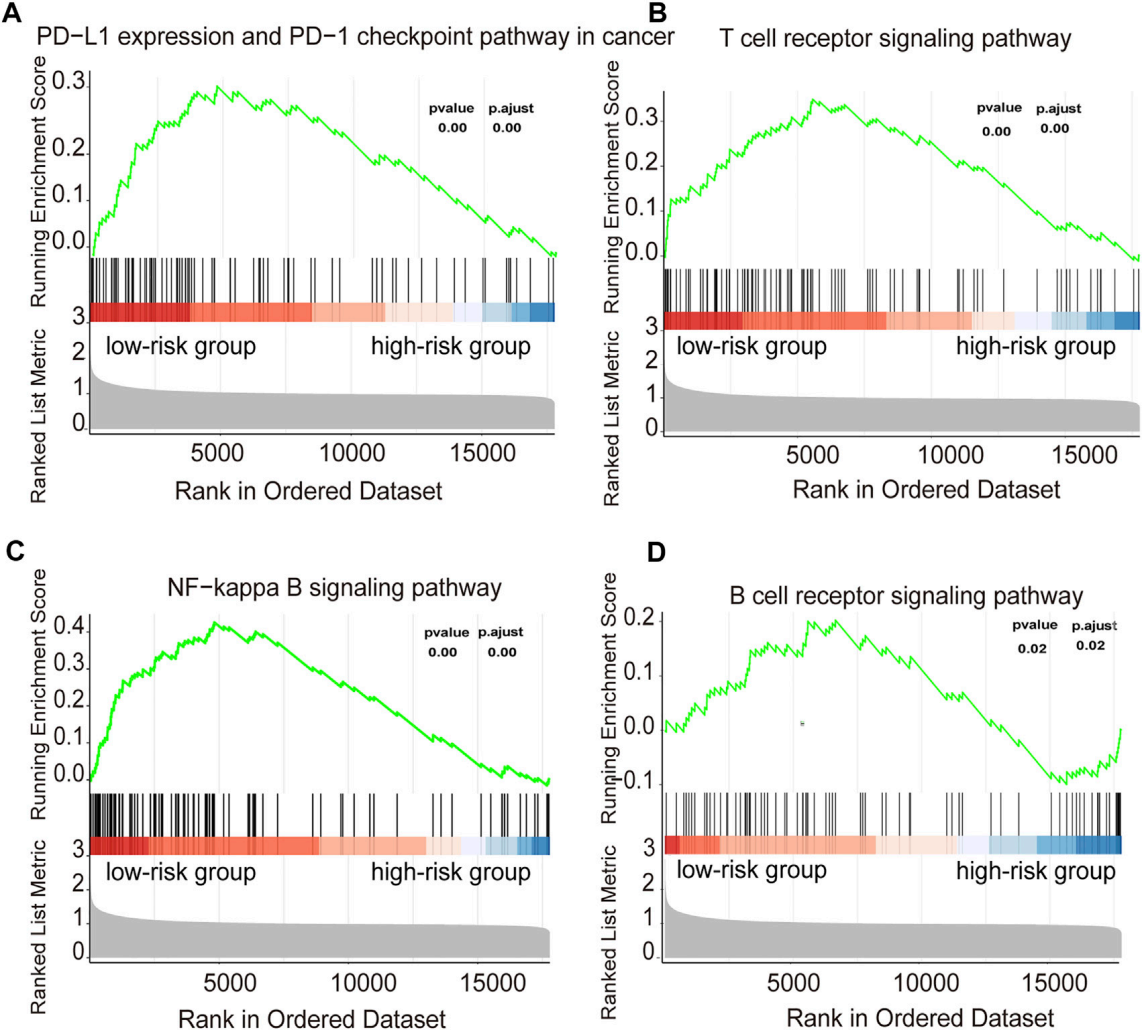
Functional annotation of the two risk subtypes in training cohort **(A–D)** Enriched gene pathways/functions in distinct risk groups from the DLBCL cohort were assessed by using the KEGG algorithm.

The GSEA enrichment analysis show that many of these pathways are related to the immune response in DLBCL both in training and validation cohorts (Figure 8, Supplementary Figure S5). In the KEGG analysis of the high- and low-risk groups, several immune-related gene sets, including T cell receptor signaling pathway, B cell receptor signaling pathway, NF−kappa B signaling pathway, PD−L1 expression and PD−1 checkpoint pathway, were enriched in the low-risk group with *p* adjust <0.05 as the cut-off threshold both in training and validation cohorts. These results indicated that the signature derived from the immune infiltration classification could represent similar biological differences in DLBCL.

### Prognostic Value of the Established Signature

**FIGURE 9:**
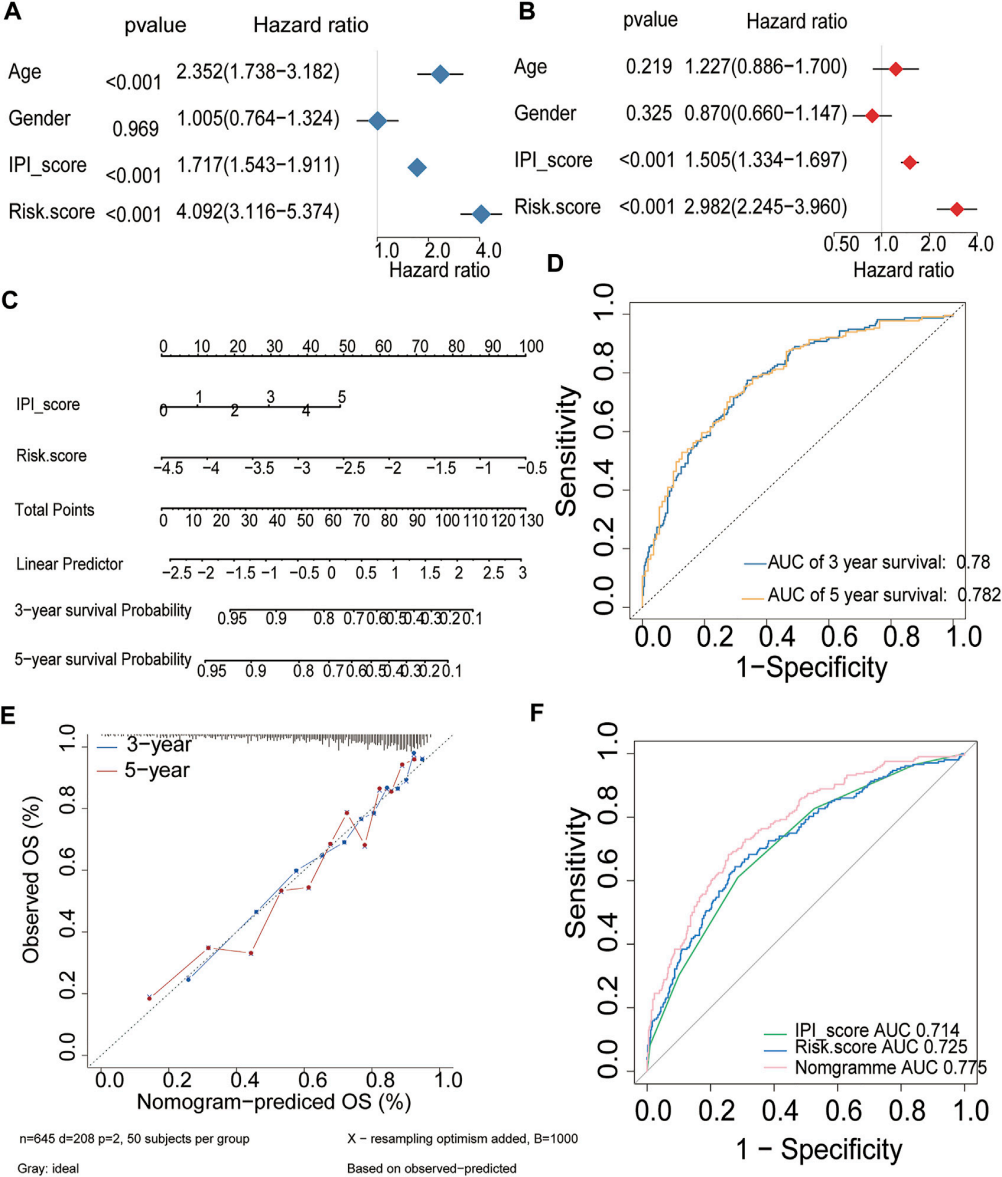
Prognostic value of the established signature in training cohort **(A,B)** Univariate and multivariate Cox regression analyses of the association between clinicopathological factors and OS of DLBCL patients **(C)** The nomogram of IPI score and the risk score **(D)** ROC curve analysis for OS prediction by the nomogram **(E)** Calibration curve of the nomogram for predicting the OS rates of DLBCL patients **(F)** ROC curves and AUCs for evaluating the prediction accuracy of the nomogram, immune risk score and IPI score.

Uni- and multivariable analyses were performed to evaluate the correlation between the risk score and survival both in training and validation cohorts (Figure 9, Supplementary Figure S6). Univariate Cox regression analysis showed that the risk score, age and IPI were both important risk factors for DLBCL (HR > 1, *p* < 0.001) (Figure 9A, Supplementary Figure S6A). Additionally, multivariate Cox analysis revealed that the risk score and IPI were independent prognostic factors for DLBCL survival (HR > 1, *p* < 0.001) (Figure 9B, Supplementary Figure S6B). Both univariate and multivariate Cox regression analyses indicated that the HR value of the risk score has an equally important role with IPI, and even greater than that of the IPI, which is currently considered to be the best prognostic criterion for DLBCL. We next constructed a survival prediction nomogram comprising the risk score and IPI to predict 3- and 5-years OS (Figure 9C, Supplementary Figure S6C). Calibration curves for the probability of 3- and 5-years survival showed good agreement between predictions and observations, which indicated that the established nomogram was reliable in predicting the prognosis of DLBCL (Figures 9D,E, Supplementary Figures S6D,E). Next, an ROC curve was plotted to observe the predicted value of the nomogram with or without the risk score. Interestingly, we noticed that the nomogram risk score AUC was 0.775, which was better than the IPI AUC (0.714) and risk score AUC (0.725) in training cohorts (Figure 9F). And in validation cohorts, the results also show that nomogram risk score AUC was better than the IPI and risk score AUC. These data suggest that the nomogram was a better predictor for a poor prognosis than IPI in DLBCL.

## Discussion

A better understanding of the relationship between the TME and lymphoma cells is urgently needed to improve the efficacy of DLBCL therapy and prognosis. Here, we describe a new stratification model for DLBCL patients with different immune cell infiltration. To improve its application in the clinic, a prognostic signature was constructed based on the different expression of mRNA from these two subgroups. Then, a novel risk score was developed based on the signature. The risk score was a promising biomarker to predict the prognosis, molecular and immune characteristics, and the immune benefit from immune checkpoint inhibitors (ICI) therapy in DLBCL.

The tumor microenvironment (TME) is an integrative component of most tumors including lymphomas. Its main cellular components are reactive lymphocytes, macrophages, fibroblasts, endothelial cells and dendritic cells as well as various cytokines, growth factors, and chemokines (Scott and Gascoyne, 2014). There is a much closer relationship between TME cells and lymphoma than other cancers because lymphoma cells themselves stem from lymphocytes (Menter et al., 2021). In contrast to Hodgkin lymphoma (HL) and indolent B‐cell lymphomas, the role of the microenvironment in aggressive B‐cell lymphomas is still a matter of debate. DLBCL was regarded as “effacement” of the TME or “non‐inflamed” lymphomas. Li, L. et al. showed the complexity of the TME in DLBCL; these cases with high T‐cell infiltrates showed an overall better prognosis, while the prognosis of T‐cell‐rich DLBCL cases was worsened by a high proportion of PD‐1‐positive T‐cells (Li et al., 2019). Xu-Monette, Z. Y. et al. demonstrated the negative impact of PD‐1‐ and CD8‐positive T‐cells in cases of PD‐L1 positivity of lymphoma cells, and a high proportion of PD‐L1‐positive TME macrophages was linked to inferior outcomes (Xu-Monette et al., 2019). In contrast to PD‐1 and PD‐L1, CTLA4 was associated with better overall survival, although it was only expressed in a minority of cases. A study by Leivonen, S. K. et al. showed that patients with high T-cell infiltration had a better response to rituximab-based immunochemotherapy (Leivonen et al., 2019). Steen CB. et al. and Kotlov N et al. highlight the heterogeneity of the tumor microenvironment in DLBCL (Kotlov et al., 2021; Steen et al., 2021). Given its complexity in DLBCL, our study utilized artificial intelligence and computational diagnostics to provide new insights into the role of the TME in DLBCL and to identify a prognostic biomarker for immunotherapy.

We first separated DLBCL patients into the Immunity-L and Immunity-H groups. The reliability of this classification was demonstrated by the obviously different gene expression levels of MHC, IAP, and ICP. Patients in the Immunity-L group exhibited decreased expression at these sites. In addition, we observed a higher proportion of activated B cells in Immunity-L group tumors, while tumors in the Immunity-H group displayed higher levels of activated CD4+/CD8+ T cells, T follicular helper cells and natural killer T cells, which contribute to the immune control of the malignant clone and impact the efficacy of chemoimmunotherapy. In addition, GO analyses found that Immunity-H group tumors showed higher expression of genes involved in the immune pathway.

Then, we developed an immune-related genetic prognostic index based on the different subgroups and analyzed its role in discriminating different molecular and immune characteristics, responses to different single drugs and outcomes of DLBCL. The comprehensive results showed that the high-risk group was correlated with lower immune infiltration, more aggressive phenotypes, lower overall survival and more sensitive to lenalidomide. In contrast, a low-risk group score was associated with higher immune infiltration, less aggressive phenotypes, better overall survival and more likely to benefit from PD-1/PD-L1 inhibitors.

Next, we explored the relationship between the risk score and the response to different single drugs. Through a prediction analysis, we found that patients in the high-risk group were more sensitive to lenalidomide. It is an immunomodulatory agent that exerts tumor toxicities by altering the tumor microenvironment (Garciaz et al., 2016). Moreover, lenalidomide has the ability to penetrate the blood–brain barrier. Combined with our results, patients in the high-risk group were also more sensitive to methotrexate and cytarabine, which are standard drugs for reducing the risk of CNS relapses. Thus, we speculate that the addition of lenalidomide treatment to chemotherapy may reduce the risk of CNS relapses in high-risk group patients, which needs to be validated in the future. Given the unimpressive efficacy of PD1/PD-L1 blockade in DLBCL, it is especially urgent to identify DLBCL patients who might benefit from anti-PD-1/PD-L1 immunotherapy. The KEGG enrichment analyses indicated that PD-L1 expression and the PD-1 checkpoint pathway were enriched in the low-risk group, which implying that patients in the low-risk group could benefit more from PD-1/PD-L1 inhibitors than high-risk patients. Together, our data provided predictive value for testing different immunotherapy in DLBCL patients.

Currently, IPIs are commonly used in clinical practice to predict the outcomes of DLBCL patients. However, the predictions are not as accurate as expected. Sixteen immune-related genes were used to establish our prognostic signature to improve the accuracy of 1-, 3-, and 5-years OS prediction. Meanwhile, we found that the risk score could separate the patients into two subgroups not only for patients in the IPI ^high^ group. This suggested that DLBC patients in the same IPI group may have distinct pathogenic mechanisms and outcomes and may receive individualized therapy. Among these 16 genes, IL-2-inducible tyrosine kinase (ITK) belongs to the Tec family of kinases and is mainly expressed in T cells (Lechner et al., 2020). Some studies have focused on ITK as a key target for drug design. Recent studies have shown that CDC20 and PTGDS were able to predict overall survival (OS) in DLBCL, which was consistent with our study (Sun et al., 2019). Wang, H.et al. reported that FBN1 promotes DLBCL cell migration by activating the Wnt/β-catenin signaling pathway and regulating TIMP1(Wang et al., 2020). Our studies found that these genes were related to overall survival. Further experiments are needed to verify the role of immune genes in DLBCL. Finally, ROC curves and calibration curves were used to construct a nomogram with precise accuracy for OS prediction. The predictive performance of the established prognostic signature was better for DLBCL patient outcomes than IPI. The nomogram, combined with the risk score and the IPI, would greatly improve the clinical prediction of DLBCL patient outcomes.

In conclusion, our study divided DLBCL patients into two subgroups based on immune infiltration. Then, we constructed an immune-related signature that was closely associated with prognosis, clinical characteristics, the immune response, and the tumor microenvironment. The prognostic signature revealed a significantly improved OS predictive ability compared to traditional prediction methods. However, one of the limitation of our study is that it was completed by using online datasets. Meanwhile, given the advent of single-cell RNA sequencing, another limitation is that we don’t include these data. Further analysis of single-cell RNA sequencing combined with clinical and basic experiments are needed to verify our results.

## Data Availability

Publicly available datasets were analyzed in this study. This data can be found here: https://portal.gdc.cancer.gov/, https://www. ncbi.nlm.nih.gov/geo/. The request for the R code used in all analyses should be directed to the corresponding authors.
