# Supplementary material for: Immunophenotypic Landscape and Prognosis-Related mRNA Signature in Diffuse Large B Cell Lymphoma
Source: Front Genet. 2022 Jun 8;13:872001. doi: 10.3389/fgene.2022.872001 (PMC9214219; doi:10.3389/fgene.2022.872001)
Supplement: Supplementary file 1 [file DataSheet1.PDF]

Supplementary Table 1:

Main Clinical Features of training cohorts and validation cohorts

#: the clinical information is incomplete in validation cohort.

	training cohort	#validation cohort
**sex**		
**female**	**330(45)**	**164(44)**
**male**	**408(55)**	**211(56)**
**Age**		
**＜60**	**320(43)**	**169(43)**
**≥60**	**418(57)**	**223(57)**
**stage**		
**I or II**	**316(44)**	**177(46)**
**III or IV**	**395(56)**	**208(54)**
**IPI**		
**0-2**	**393(61)**	**203(70)**
**3-5**	**252(39)**	**86(30)**
**Extranodal involvement**		
**yes**	**296(58)**	**142(39)**
**no**	**218(42)**	**219(61)**
**COO**		
**ABC**	**282(48)**	**162 (49)**
**GCB**	**311(52)**	**169(51)**
**R-CHOP response**		
**CR**	**354(75)**	
**PR**	**72(15)**	
**PD/SD**	**44(9)**	

Supplementary Table 2: Main Clinical Features of DLBCL based on two clusters

*p < 0.05;#: there were two patients didn't have exact value.

	Immunity-L	Immunity-H	**P value**
**sex**			**0.867**
**female**	**163(45)**	**167(44.4)**	
**male**	**199(55)**	**209(55.6)**	
**Age**			**0.075**
**＜60**	**145(40.1)**	**175(46.5)**	
**≥60**	**217(59.9)**	**201(53.5)**	
**stage**			**0.987**
**I or II**	**157(44.5)**	**159(44.4)**	
**III or IV**	**196(55.5)**	**199(55.6)**	
**IPI**			***0.011**

**0-2**	**181(56)**	**212(65.8)**	
**3-5**	**142(44)**	**110(34.2)**	
**#Extranodal involvement**			**0.726**
**yes**	**140(56.7)**	**156(58.2)**	
**no**	**107(43.3)**	**1112(4c1.8)**	
**COO**			**0.407**
**ABC**	**140 (45.9)**	**142(49.3)**	
**GCB**	**165(54.1)**	**146(50.7)**	
**R-CHOP response**			**0.738**
**CR**	**166(73.8)**	**188(76.7)**	
**PR**	**36(16)**	**36(14.7)**	
**PD/SD**	**23(10.2)**	**21(8.6)**	

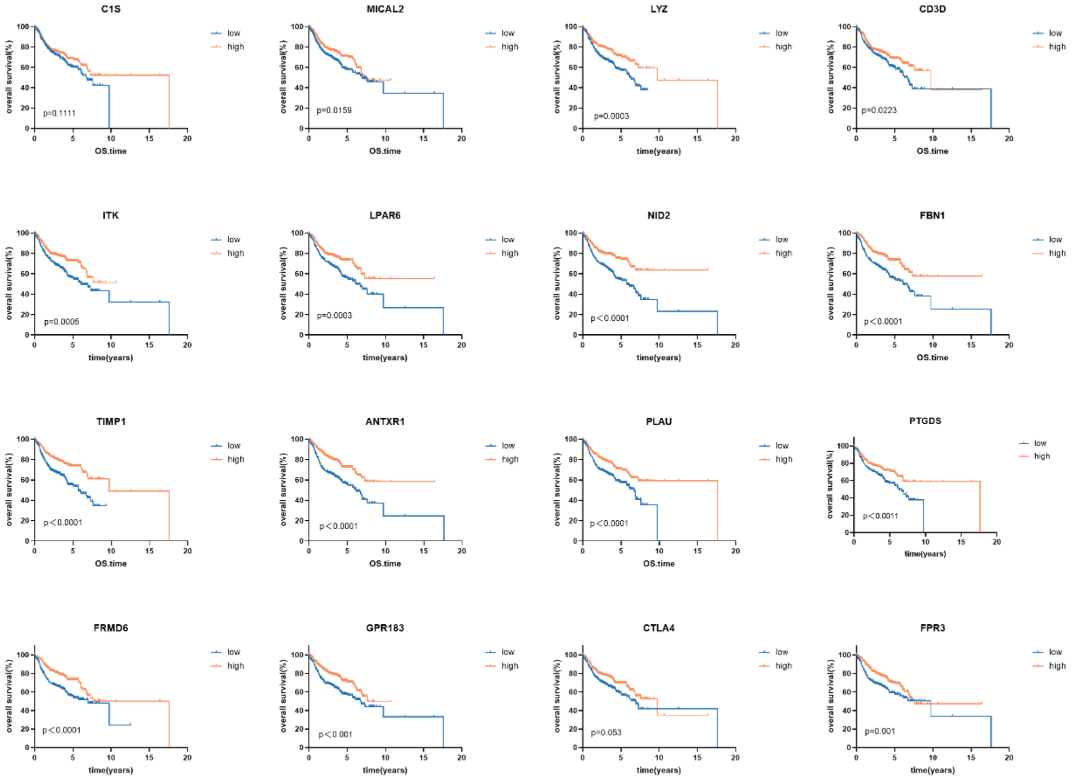

Supplementary Figure1: Kaplan–Meier analysis of the 16 immune related gene in training cohort

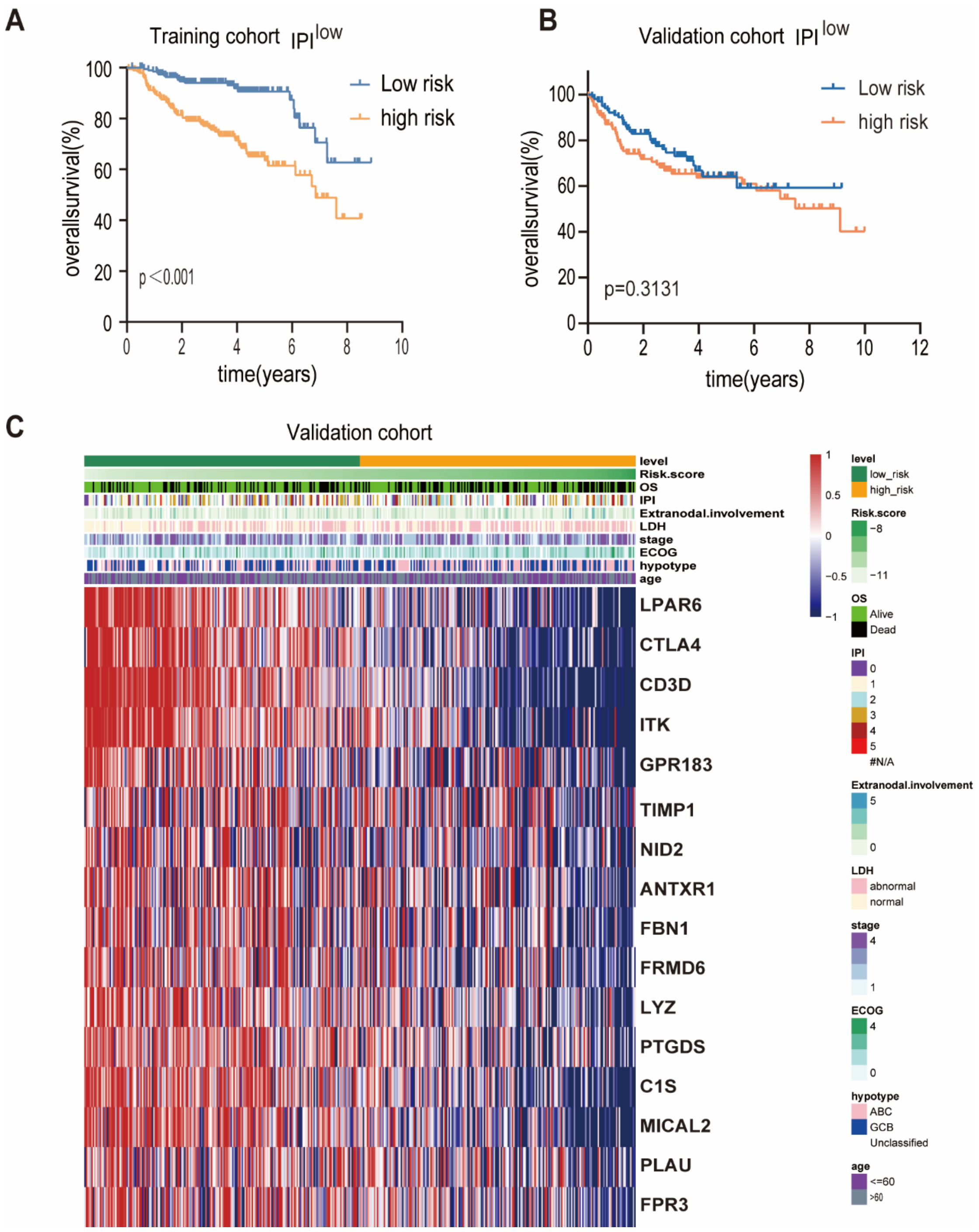

Supplementary Figure2: Assessment of a classification-related prognostic signature for DLBCL

(A-B) Kaplan–Meier analysis of the high versus low risk score in the IPI ^{low} group for the training and validation cohort.

(C)The differential expression of the 16 genes in the high- and low-risk groups in the validation cohort.

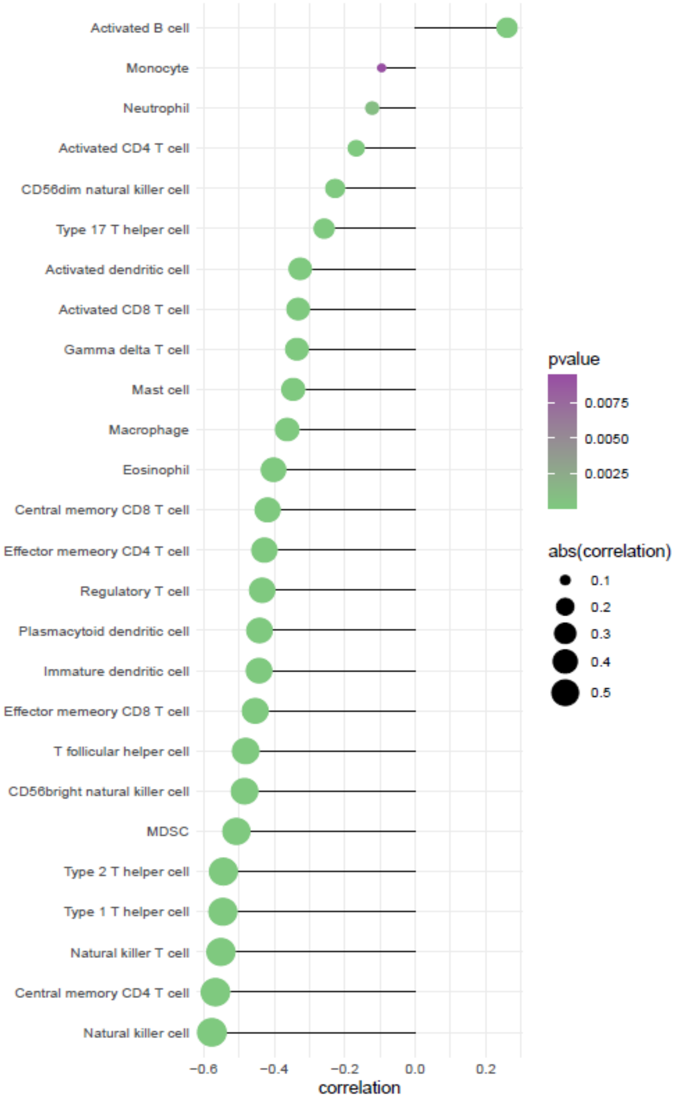

Supplementary Figure3: The Gene Ontology functional enrichment analysis of the differentially expressed genes based on the immune risk score in training cohort. Low immune risk score group was used as control.

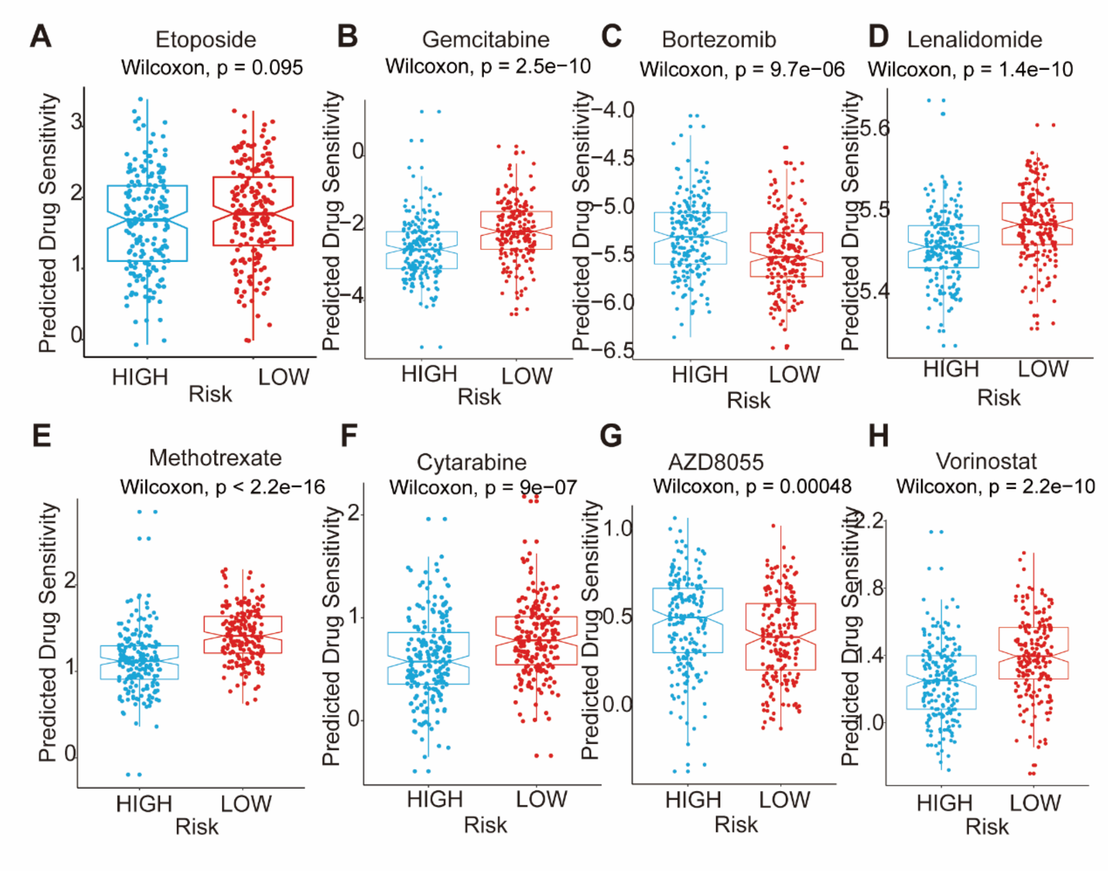

Supplementary Figure4:

The predicted sensitivity of chemotherapeutic agents with mRNA signature in validation cohort

(A) Etoposide, **(B)** Gemcitabine, **(C)** Bortezomib, **(D)** Lenalidomide, **(E)** Methotrexate, **(F)** Cytarabine, **(G)** AZD8055, **(H)** Vorinostat.

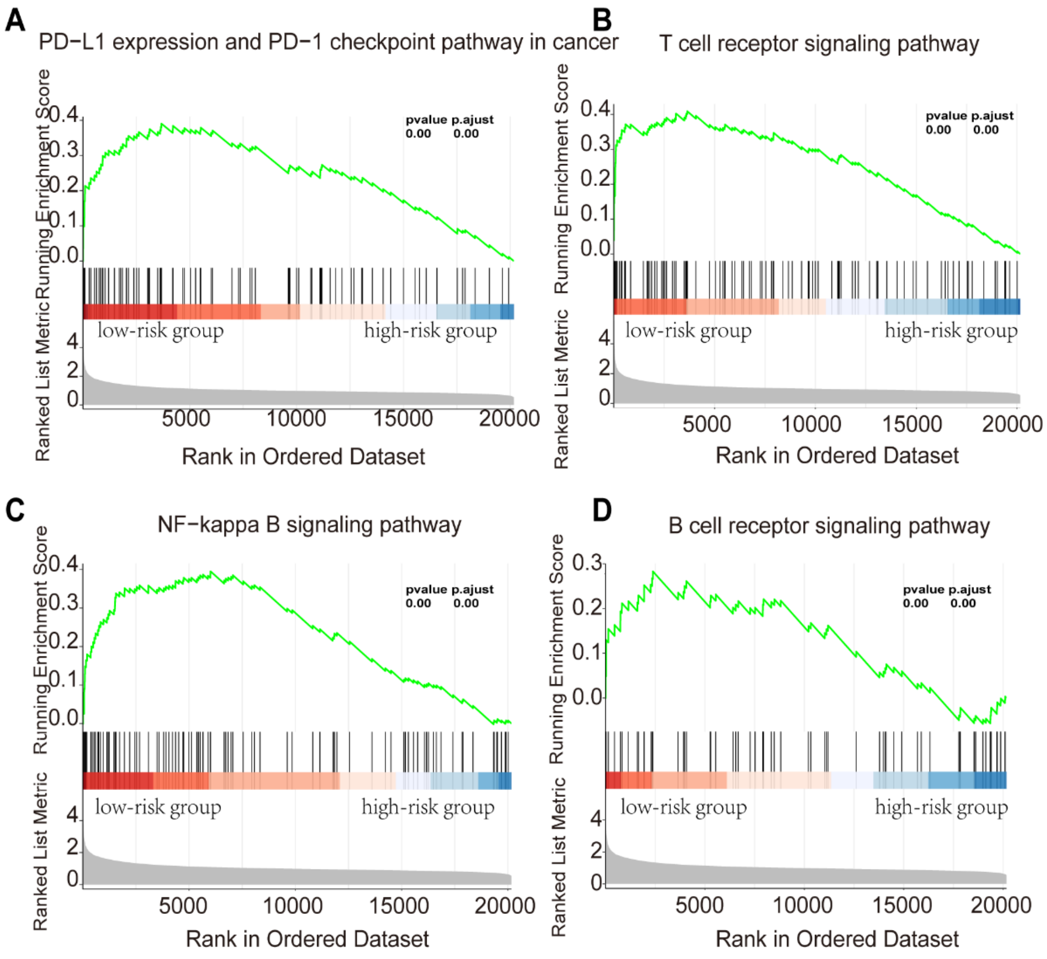

Supplementary Figure5: Functional annotation of the two risk subtypes in validation cohort

(A-D) Enriched gene pathways/functions in distinct risk groups from the DLBCL cohort were assessed by using the KEGG algorithm

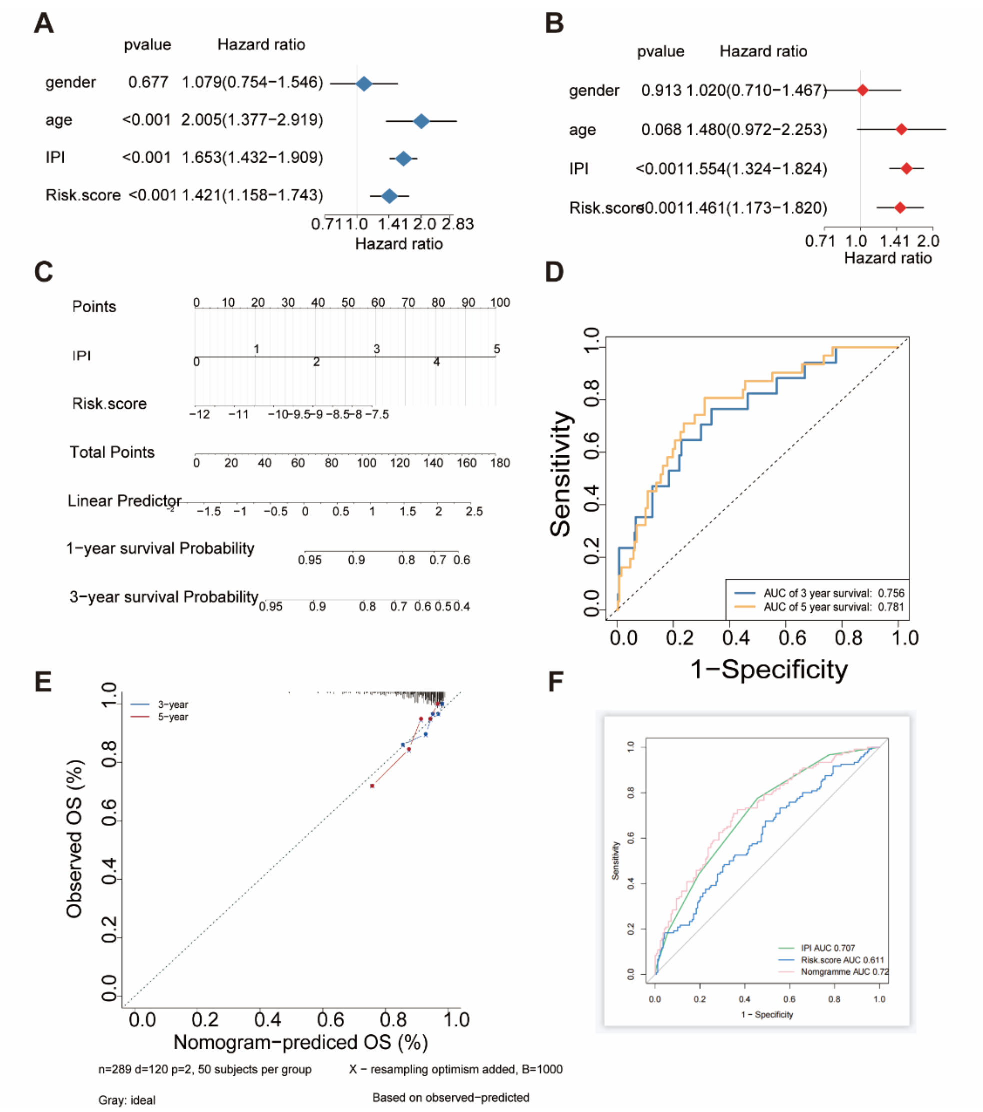

Supplementary Figure6:

Prognostic value of the established signature in training cohort

(A-B) Univariate and multivariate Cox regression analyses of the association between clinicopathological factors and OS of DLBCL patients.

(C) The nomogram of IPI score and the risk score.

(D) ROC curve analysis for OS prediction by the nomogram.

(E) Calibration curve of the nomogram for predicting the OS rates of DLBCL patients

(F) ROC curves and AUCs for evaluating the prediction accuracy of the nomogram, immune risk score and IPI score.
